# Betacellulin regulates gap junction intercellular communication by inducing the phosphorylation of connexin 43 in human granulosa-lutein cells

**DOI:** 10.1186/s13048-023-01185-3

**Published:** 2023-05-25

**Authors:** Yuxi Li, Hsun-Ming Chang, Yu-Wen Sung, Hua Zhu, Peter C. K. Leung, Ying-Pu Sun

**Affiliations:** 1 Center for Reproductive Medicine, The First Affiliated Hospital of Zhengzhou University, 40, Daxue Road, Zhengzhou, 450052 Henan China; 2 Department of Obstetrics and Gynecology, BC Children’s Hospital Research Institute, University of British Columbia, Room 317, 950 West 28th Avenue, Vancouver, BC V5Z 4H4 Canada; 3 Henan Key Laboratory of Reproduction and Genetics, The First Affiliated Hospital of Zhengzhou University, Zhengzhou, China; 4 Henan Provincial Obstetrical and Gynecological Diseases (Reproductive Medicine) Clinical Research Center, The First Affiliated Hospital of Zhengzhou University, Zhengzhou, China; 5 Reproductive Medicine Center, Department of Obstetrics and Gynecology, China Medical University Hospital, Taichung, Taiwan

**Keywords:** Betacellulin, Connexin 43, Gap junction intercellular communication, Human granulosa cells, PKC

## Abstract

**Background:**

The gap junction protein, connexin 43 (Cx43) is highly expressed in human granulosa-lutein (hGL) cells. The phosphorylation of certain amino acid residues in the Cx43 protein has been shown to be related to a decline in gap junction intercellular communication (GJIC), which subsequently affects oocyte meiotic resumption. As a member of the epidermal growth factor (EGF) family, betacellulin (BTC) mediates luteinizing hormone (LH)-induced oocyte maturation and cumulus cell expansion in mammalian follicles. Whether BTC can regulate Cx43 phosphorylation, which further reduces Cx43-coupled GJIC activity in hGL cells remains to be determined.

**Methods:**

Immortalized human granulosa cells (SVOG cells) and primary human granulosa-lutein cells obtained from women undergoing in vitro fertilization in an academic research center were used as the study models. The expression levels of Cx43 and phosphorylated Cx43 were examined following cell incubation with BTC at different time points. Several kinase inhibitors (sotrastaurin, AG1478, and U0126) and small interfering RNAs targeting EGF receptor (EGFR) and receptor tyrosine-protein kinase 4 (ErbB4) were used to verify the specificity of the effects and to investigate the molecular mechanisms. Real-time-quantitative PCR and western blot analysis were used to detect the specific mRNA and protein levels, respectively. GJIC between SVOG cells were evaluated using a scrape loading and dye transfer assay. Results were analyzed by one-way analysis of variance.

**Results:**

The results showed that BTC induced the rapid phosphorylation of Cx43 at serine368 without altering the expression of Cx43 in primary and immortalized hGL cells. Additionally, using a dual inhibition approach (kinase inhibitors and siRNA-based expression knockdown), we demonstrated that this effect was mainly mediated by the EGFR but not the ErbB4 receptor. Furthermore, using a protein kinase C (PKC) kinase assay and a scrape-loading and dye transfer assay, we revealed that PKC signaling is the downstream signaling pathway that mediates the increase in Cx43 phosphorylation and subsequent decrease in GJIC activity in response to BTC treatment in hGL cells.

**Conclusions:**

BTC promptly induced the phosphorylation of connexin 43 at Ser368, leading to decreased GJIC activity in hGL cells. The BTC-induced cellular activities were most likely driven by the EGFR-mediated PKC-dependent signaling pathway. Our findings shed light on the detailed molecular mechanisms by which BTC regulates the process of oocyte meiotic resumption.

## Background

Belonging to the epidermal growth factor (EGF) family, betacellulin (BTC) is widely expressed in the human digestive system (pancreas, kidney, liver, and colon) and reproductive system (testis and ovary) [1–3]. Emerging evidence indicates a rapid increase in the expression and release of amphiregulin (AREG), BTC and epiregulin (EREG) induced by a luteinizing hormone (LH) surge, and these intrafollicular factors are essential for cumulus expansion and oocyte meiotic resumption in mammalian ovarian follicles [4, 5]. In 2004, Park et al. first reported that the LH surge induced rapid expression of the EGF family members (including AREG, EREG, and BTC) in mouse preovulatory follicles. Additionally, incubation of BTC effectively promoted the resumption of oocyte meiosis characterized by germinal vesicle breakdown (GVBD), expansion of cumulus-oophorus complex (COC), and oocyte maturation [2]. Ashkenazi et al. further verified that LH promoted the transient expression of BTC mRNA with a maximal level at 3 h followed by a marked decrease level at 9 h. They also confirmed that BTC stimulated the resumption of meiosis in follicle-enclosed oocytes in rats [5]. Furthermore, previous studies also indicated that the addition of BTC to cultured follicles stimulated the expression of several genes associated with cumulus expansion and ovulation, such as *Has2* and *Ptgs2* [6]. In addition to its effects on follicular development, BTC has been shown to participate in embryo implantation in mice [7, 8]. The epidermal growth factor receptor (EGFR) family, also known as the receptor tyrosine-protein kinase (ErbB) receptor family, includes 4 receptor tyrosine kinases (epidermal growth factor receptor or EGFR/ErbB1/HER1, ErbB2/HER2, ErbB3/HER3, and ErbB4/HER4). In many cell types, BTC exerts its cellular effects by binding to ErbB1 and ErbB4 homodimers with high affinity and all possible combinations of ErbB heterodimers [9, 10]. Upon ligand‒receptor binding, various intracellular signaling pathways (including MAPK, PI3K/AKT, and JNK) can be induced by BTC [11, 12].

Before ovulation, an oocyte remains meiotic arrest at prophase I stage, and it is affected by intracellular cAMP produced by cumulus cells and transmitted through gap junctions [13]. Moreover, the intraoocyte levels of cAMP can be regulated by cGMP produced by granulosa cells and cumulus cells [14]. In this regard, the gap junction-mediated cell‒cell interactions are critical for normal ovulation and oocyte maturation [15]. Gap junction channels permit small molecules, inorganic ions, and second messengers to be translocated through adjacent cells, a critical mechanism of intercellular communication. Each cell contains a cylindrical organelle (hemichannel or connexon) in the plasma membrane, and a hemichannel in one cell interacts with a hemichannel in an adjacent cell to form a complete gap junction channel [16]. A connexon is formed by a hexamer of protein subunits named connexins [16]. Among the numerous members of the connexin family, connexin 43 (Cx43, also known as GJA1) is a major mediator of communication between granulosa cells, which are involved in the regulation of follicular development and oocyte maturation [17]. In the cumulus cells of many mammals, including rats, pigs, and cattle, decreased expression of Cx43 is associated with oocyte meiotic resumption [18–20]. Several factors, including gonadotropins [21], steroid hormones [22], and LH, [23] have been shown to affect the gap junction intercellular communication (GJIC) by regulating the Cx43 expression and phosphorylation [21–23]. As a phosphoprotein, Cx43 can be phosphorylated in the carboxyl-terminus, and phosphorylation at different sites in Cx43 protein has been correlated with differential changes in gap junction communication between cells. For instance, the phosphorylation of Cx43 at Ser325, Ser328, Ser364/365, and Ser373 results in an increase in GJIC, whereas the phosphorylation of Cx43 at Ser255, Ser262, Ser279/282, and Ser368 leads to a decrease in GJIC [24]. In rat primary granulosa cells, follicle-stimulating hormone (FSH) enhances Cx43-mediated GJIC via phosphorylation of different sites (Ser365, Ser368, Ser369 and Ser373) in the Cx43 protein [25], whereas LH-induced MAPK-mediated phosphorylation of Cx43 suppresses GJIC between follicular somatic cells, contributing to oocyte meiotic resumption in mouse ovaries [23]. Eventually, the LH surge-induced resumption of meiosis promotes the oocyte to enter the second cycle of meiosis, which is known as oocyte maturation at the time of ovulation [26]. In mice, the administration of an inhibitor of MAPK activation that blocked gap junction closure in response to LH prevented oocyte meiosis resumption, leading to failure of oocyte maturation [23].

Given the critical role played by LH-induced ovarian follicle-derived EGF family factors in the regulation of oocyte maturation [2, 27] and because the termination of Cx43-mediated GJIC contributes to oocyte meiotic resumption, we aimed to investigate whether BTC (an EGF family factor) can regulate phosphorylation of Cx43 protein and subsequently affects GJIC in human granulosa cells.

## Results

### BTC induced the phosphorylation of Cx43 in SVOG and primary hGL cells

**Fig. 1:**
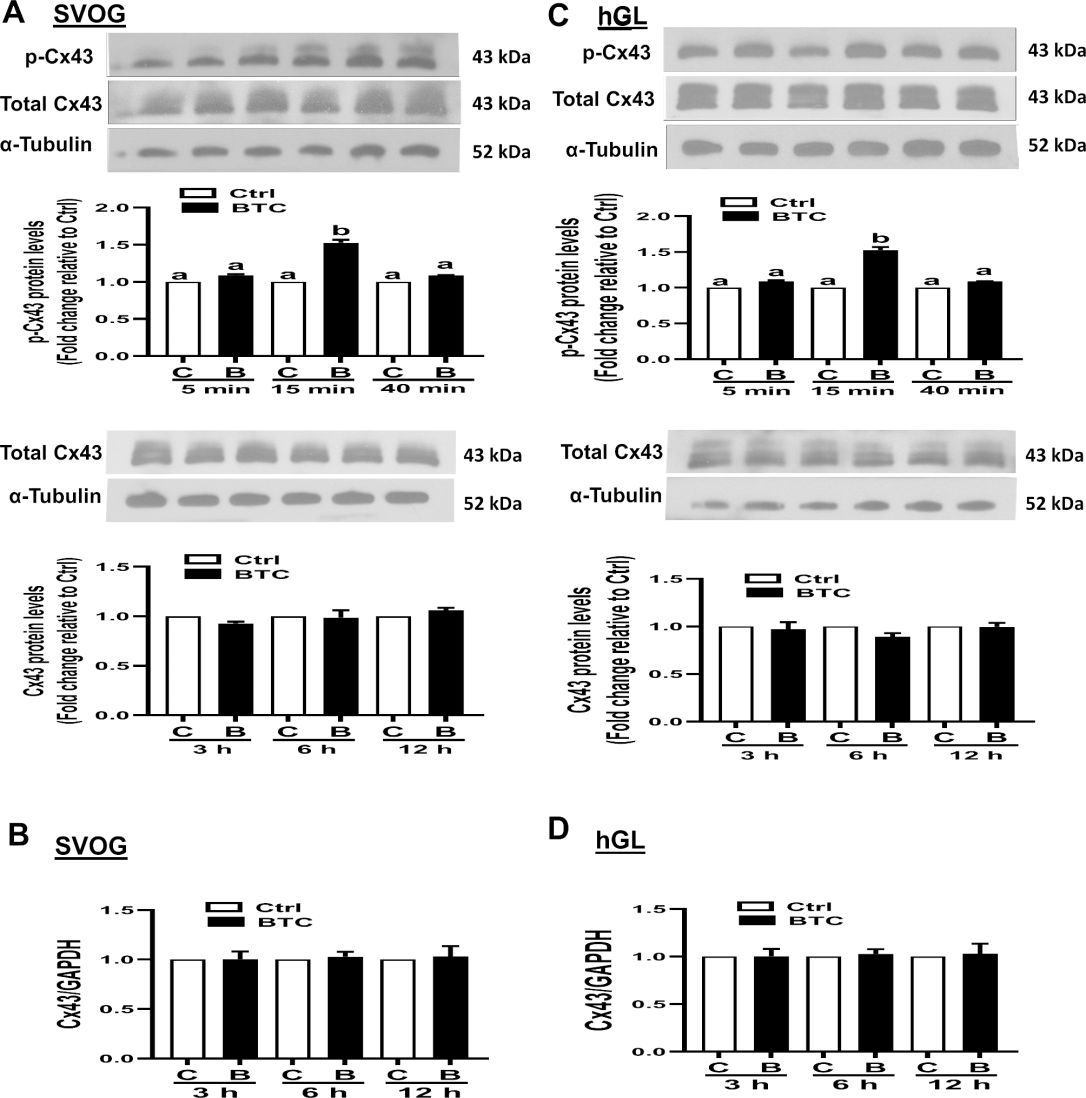
Effects of betacellulin (BTC) on Cx43 phosphorylation and expression in SVOG and primary human granulosa-lutein (hGL) cells. (A and C) SVOG (A) or hGL (C) cells were treated with 50 ng/ml BTC for different time durations (5, 15, or 40 min), and the levels of phosphorylated Cx43 protein were examined via Western blot analysis (quantified data were normalized to those of α-tubulin). (A and C) SVOG (A) or hGL (C) cells were treated with 50 ng/ml BTC for different time durations (3, 6, or 12 h), and the protein levels of Cx43 were measured via Western blot analysis (quantified data were normalized to those of α-tubulin). (B and D) SVOG (B) or hGL (D) cells were treated with 50 ng/ml BTC for different time durations (3, 6, or 12 h), and the mRNA levels of Cx43 were examined via RT‒qPCR. Band intensities of western blot staining were measured and quantitatively analyzed using ImageJ 1.42 (NIH, U.S.A.). The experiments were performed in triplicate. Quantification graphs reflect Integrated Density Values of the treatment group divided by the Integrated Density Values of control group. The results are expressed as the mean ± SEM of at least three independent experiments. Values marked with different letters were significantly different (P < 0.05). In detail, if the letters on two columns are different (E.g., “a” vs. “b” or “b” vs. “c”), it means that the difference between the two groups is significant, on the other hand, if the letters on the column of two groups are the same (E.g., “a” vs. “a” or “b” vs. “b”), it means there is no significant difference between two groups. B and BTC, betacellulin; C and Ctrl, control; Cx43, connexin 43

To investigate the posttranslational modification effect of BTC on Cx43, we treated the SVOG immortalized cell line with 50 ng/ml BTC for different time durations (5, 15, or 40 min). The results obtained via Western blot analysis (Fig. 1A) showed that BTC increased the level of the phosphorylated Cx43 protein (at serine 368) (15 min after treatment) in SVOG cells. To confirm that the BTC-induced increase in Cx43 phosphorylation was not due to the expressional change in response to BTC, we treated SVOG cells with BTC (50 ng/ml) for 3, 6, or 12 h. The results showed that BTC treatment did not alter Cx43 expression at the protein or mRNA level (Fig. 1A and B). Furthermore, we used primary hGL cells obtained from women undergoing IVF to confirm the regulatory role played BTC on Cx43 phosphorylation. Similarly, the results showed that BTC treatment for 15 min increased the level of the phosphorylated Cx43 protein (Ser368) in primary hGL cells (Fig. 1C). However, BTC treatment did not alter the protein o mRNA expression at any time point examined (Fig. 1C and D). These results indicate that BTC can induce the phosphorylation of Cx43 in SVOG and primary hGL cells.

### EGFR but not ErbB4 mediated the BTC-induced increase in Cx43 phosphorylation in hGL cells

**Fig. 2:**
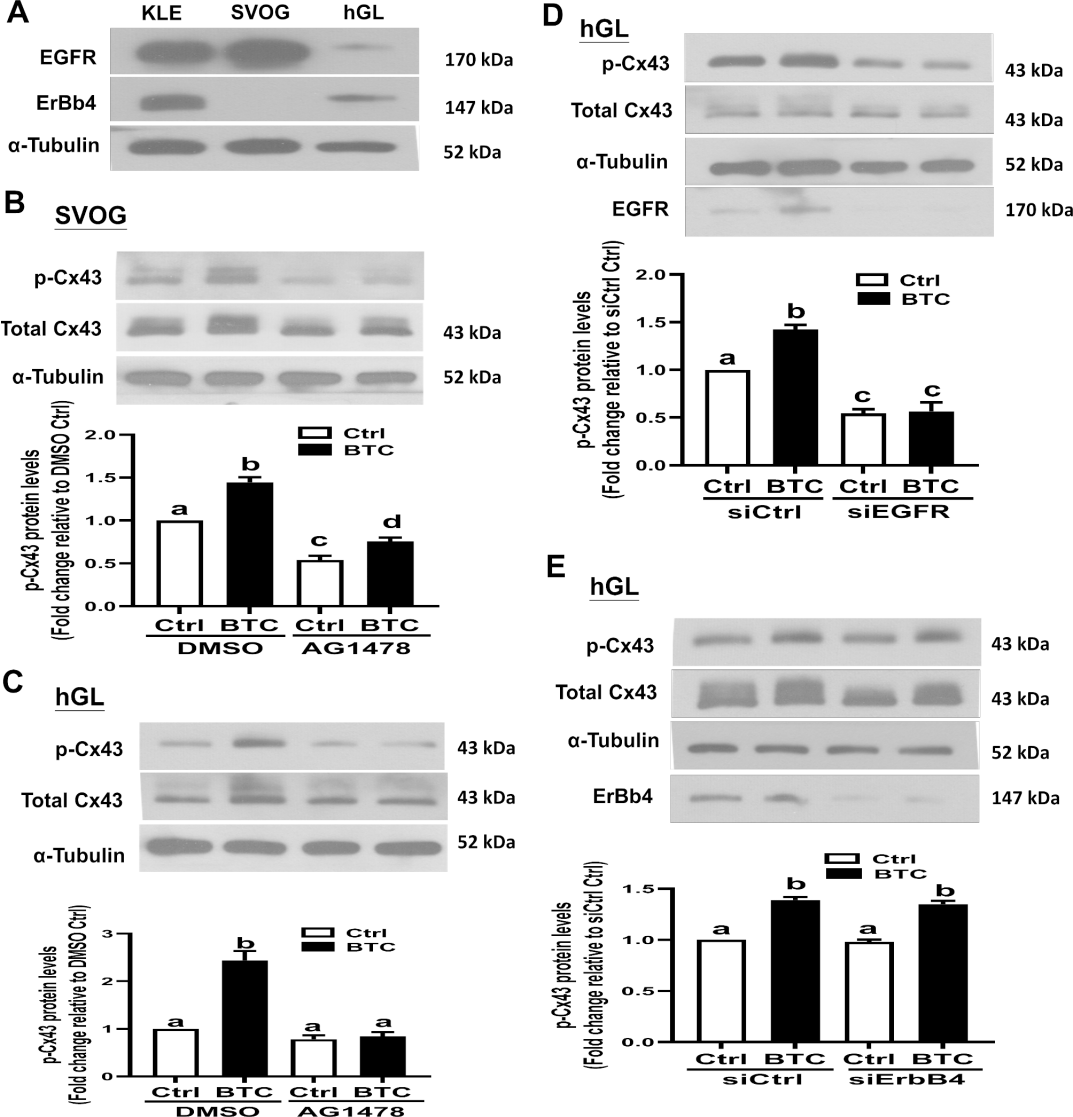
Effects of AG1478 or siRNA-mediated knockdown of EGFR (or ErBb4) on the BTC-induced increase in phosphorylated Cx43 in SVOG and hGL cells. (A) Lysates prepared with KLE, SVOG, or hGL cells were obtained, and the protein levels of EGFR and ErBb4 were measured via Western blot analysis. (B and C) SVOG (B) or hGL (C) cells were pretreated with DMSO (the vehicle control) or 10 µM sotrastaurin, and the cells were treated with 50 ng/ml BTC for an additional 15 min. The levels of phosphorylated Cx43 protein were measured via Western blot analysis. (D and E) Primary hGL cells were transfected with 25 nM siCtrl, 25 nM siEGFR (D), or 25 nM siErBb4 for 48 h, and the cells were then treated with 50 ng/ml BTC for an additional 15 min. The levels of phosphorylated Cx43 protein were measured via Western blot analysis. The results are expressed as the mean ± SEM of at least three independent experiments. Values marked with different letters were significantly different (P < 0.05)

BTC has been shown to exert cellular activities by binding to EGFR and ErbB4 receptors in many cell types [9]. (Fig. 2A). In this study, KLE cells (derived from undifferentiated endometrial carcinoma) were used as the positive controls in an analysis of the cellular expression of EGFR and ErbB4 [28]. As shown in Fig. 2A, both EGFR and ErbB4 were expressed in primary hGL cells, whereas ErbB4 was not expressed in SVOG cells. To determine which functional receptor is involved in the BTC-induced phosphorylation of Cx43 in hGL cells, a pharmacological inhibition approach was used. As shown in Fig. 2B C, pretreatment with 10 µM AG1478 (a specific inhibitor of EGFR activity) for 40 min abolished the phosphorylation of Cx43 induced by BTC in both SVOG and primary hGL cells. Additionally, specific siRNAs targeting EGFR and ErbB4 (siEGFR and siErbB4) were used to investigate the involvement of the ErbB family in the BTC-induced phosphorylation of Cx43 in primary hGL cells. Interestingly, knockdown of EGFR completely abolished the BTC-induced phosphorylation of Cx43 (Fig. 2D), whereas knockdown of ErbB4 exerted no such effect (Fig. 2E). These results indicate that EGFR but not ErbB4 is required for the phosphorylation of Cx43 in response to BTC in human hGL cells.

### Sotrastaurin (a PKC inhibitor) or AG1478 (an EGFR inhibitor) reversed the BTC-induced increase in PKC activity in SVOG cells

**Fig. 3:**
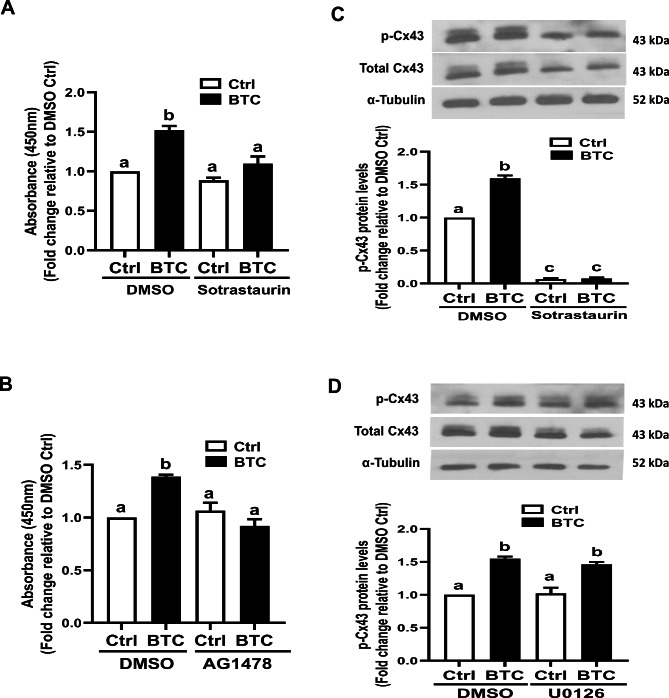
Effects of kinase inhibitors on BTC-induced increases in PKC activity and phosphorylated Cx43 in SVOG cells. (A and B) SVOG cells were pretreated with DMSO, 10 µM sotrastaurin (A) or 10 µM AG1478 (B) for 40 min, and the cells were then treated with 50 ng/ml BTC for an additional 15 min. PKC activity was measured with a PKC kinase activity kit. (C and D) SVOG cells were pretreated with DMSO, 10 µM sotrastaurin (C) or 10 µM 0126 (D) for 40 min, and the cells were then treated with 50 ng/ml BTC for an additional 15 min. The levels of phosphorylated Cx43 protein were measured via Western blot analysis. The results are expressed as the mean ± SEM of at least three independent experiments. Values marked with different letters were significantly different (P < 0.05)

**Fig. 4:**
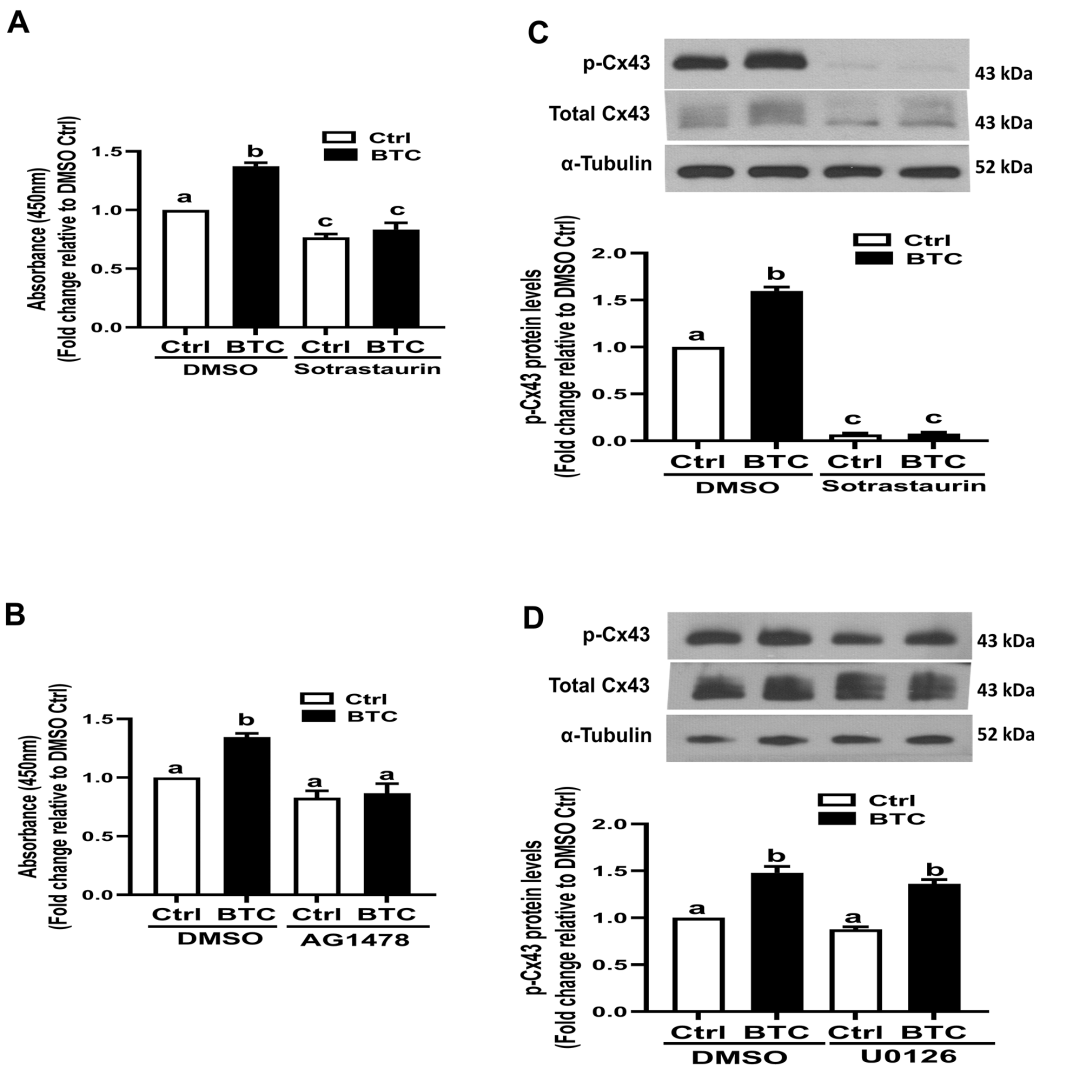
Effects of kinase inhibitors on BTC-induced increases in PKC activity and phosphorylated Cx43 in hGL cells. (A and B) Primary hGL cells were pretreated with DMSO, 10 µM sotrastaurin (A) or 10 µM AG1478 (B) for 40 min, and the cells were then treated with 50 ng/ml BTC for an additional 15 min. PKC activity was measured with a PKC kinase activity kit. (C and D) Primary hGL cells were pretreated with DMSO, 10 µM sotrastaurin (C) or 10 µM 0126 (D) for 40 min, and the cells were then treated with 50 ng/ml BTC for an additional 15 min. The levels of phosphorylated Cx43 protein were measured via Western blot analysis. The results are expressed as the mean ± SEM of at least three independent experiments. Values marked with different letters were significantly different (P < 0.05)

Previous studies have shown that various signaling pathways, including MAPK, PKC, and v-Src, are involved in the phosphorylation of different Cx43 C-terminal amino acids [29, 30]. We therefore investigated whether the MAPK or PKC signaling pathway mediates the BTC-induced phosphorylation of Cx43 in hGL cells. Using a specific PKC kinase assay, we found that PKC activity was significantly increased in response to BTC, and this effect was abolished by pretreatment with 10 µM sotrastaurin (a pan-PKC activity inhibitor) in SVOG cells (Fig. 3A). Additionally, pretreatment of SVOG cells with 10 µM AG1478 completely abolished the BTC-induced increase in PKC activity in SVOG cells (Fig. 3B). Importantly, pretreatment with 10 µM sotrastaurin completely abolished the BTC-induced phosphorylation of Cx43 in SVOG cells (Fig. 3C). However, pretreatment with 10 µM U0126 exerted no such effect (Fig. 3D). Similar results were obtained using primary hGL cells, with sotrastaurin (or AG1478, but not U0126) pretreatment completely abolishing the BTC-induced increase in PKC activity and phosphorylation of Cx43 in primary hGL cells (Fig. 4). These results indicate that the PKC signaling pathway most likely mediated BTC-induced phosphorylation of Cx43 in hGL cells.

### Localization and distribution of phosphorylated Cx43 protein in human hGL cells

**Fig. 5:**
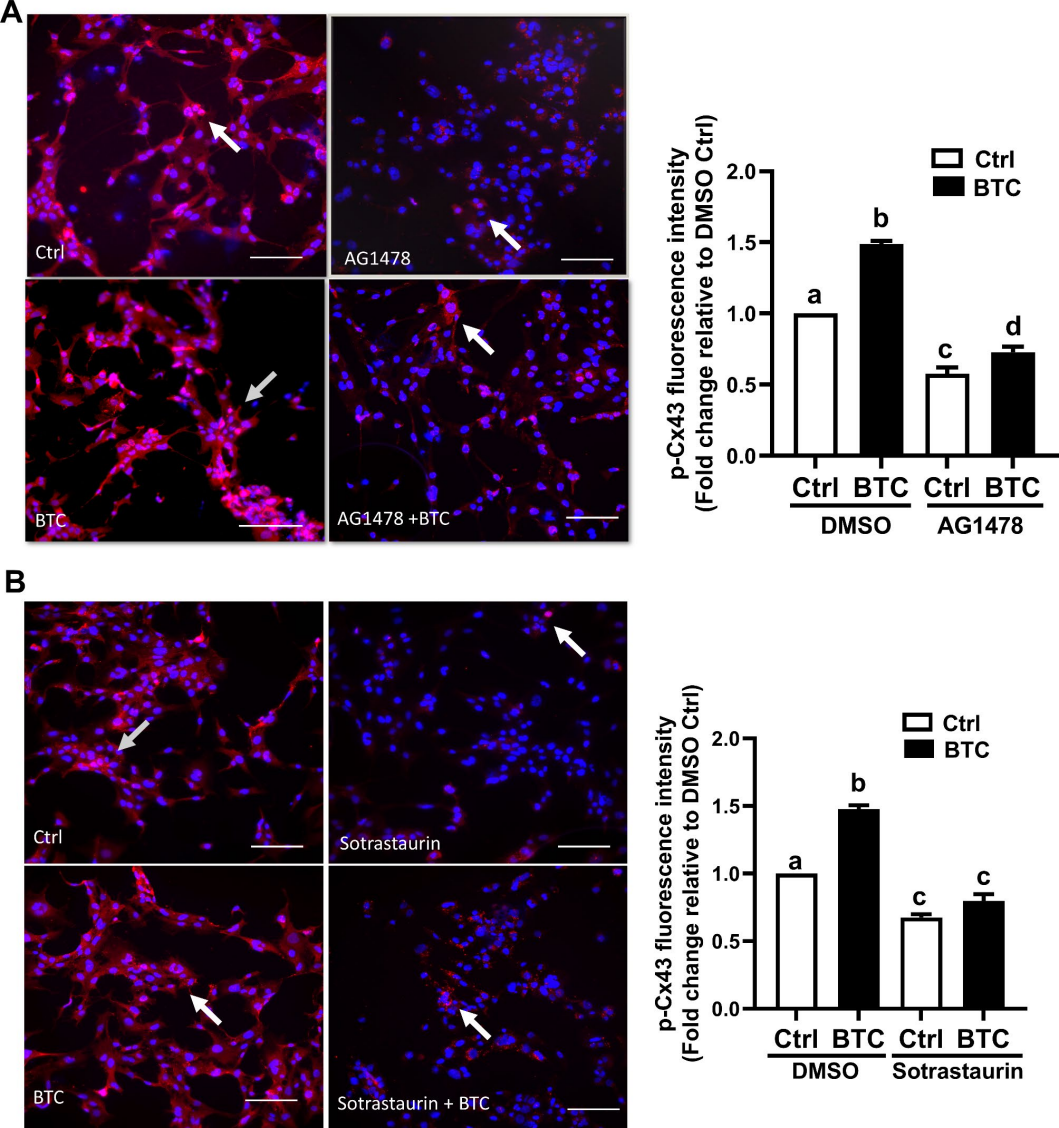
Localization and distribution of Cx43 in SVOG cells. (A and B) SVOG cells were pretreated with DMSO, 10 µM AG1478 (A) or 10 µM sotrastaurin (B) for 40 min, and the cells were then treated with 50 ng/ml BTC for an additional 15 min. The localization and distribution of Cx43 protein phosphorylation were examined by immunofluorescence microscopy. Cell nuclei were stained with DAPI. The scale bar represents 50 μm. Magnification, 400×. Quantification of the fluorescence intensities were quantified using ImageJ 1.42 (NIH, U.S.A.). The experiments were performed in triplicate. Arrow indicates the position of the phosphorylated Cx43 protein. Fluorescence intensity was calculated as fluorescence intensity of the treatment group divided by the fluorescence intensity of control group. The image background was subtracted by ImageJ software. The results are expressed as the mean ± SEM of at least three independent experiments. Values marked with different letters were significantly different (P < 0.05)

**Fig. 6:**
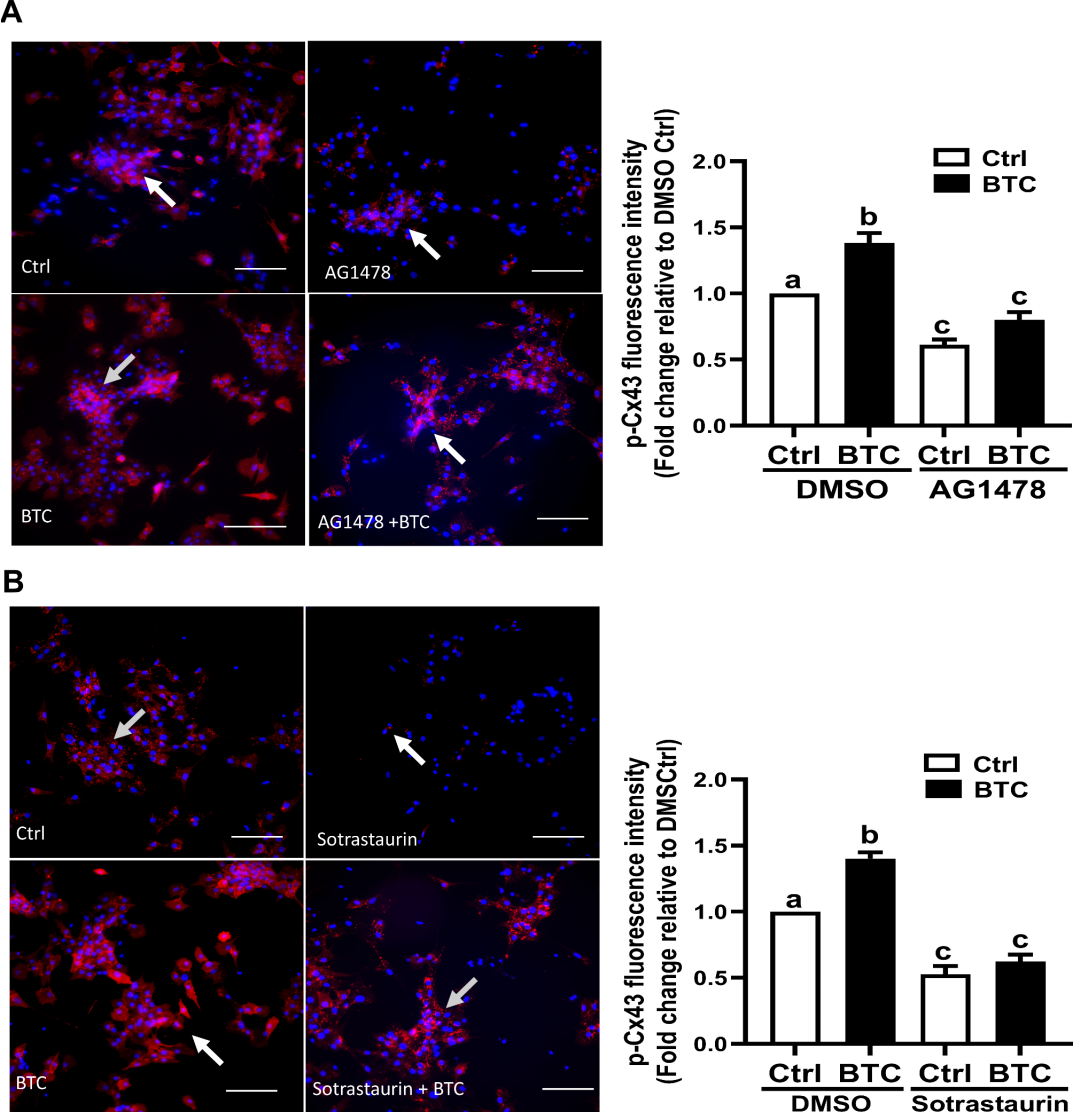
Localization and distribution of Cx43 in primary hGL cells. (A and B) Primary hGL cells were pretreated with DMSO, 10 µM AG1478 (A) or 10 µM sotrastaurin (B) for 40 min, and the cells were then treated with 50 ng/ml BTC for an additional 15 min. The localization and distribution of phosphorylated Cx43 protein were examined by immunofluorescence microscopy. Arrow indicates the position of the phosphorylated Cx43 protein. Cell nuclei were stained with DAPI. The scale bar represents 50 μm. Magnification, 400×. The results are expressed as the mean ± SEM of at least three independent experiments. Values marked with different letters were significantly different (P < 0.05)

To investigate the localization and distribution of subcellular phosphorylated Cx43 protein, we performed an immunofluorescent assay of stained SVOG and primary hGL cells. As shown in Fig. 5A, the signal of the phosphorylated Cx43 protein was detected in red fluorescent plaques assembled on cell membranes, indicating that the classical distribution of Cx43-coupled gap junctions was established between human granulosa cells. Consistent with the results of a Western blot analysis, pretreatment with 10 µM AG1478 for 40 min abolished the BTC-induced increase in Cx43 phosphorylation in SVOG cells (Fig. 5A). In addition, pretreatment with 10 µM sotrastaurin for 40 min completely abolished the BTC-induced increase in phosphorylated Cx43 in SVOG cells (Fig. 5B). Similar staining patterns were observed in primary hGL cells, showing that AG1478 or sotrastaurin completely abolished the BTC-induced increase in phosphorylated Cx43 (Fig. 6A and B). These results indicate that the BTC-induced increase in phosphorylated Cx43 can be detected between the cell-cell junction of hGL cells.

### AG1478 or sotrastaurin abolished the BTC-induced decrease in GJIC in SVOG cells

**Fig. 7:**
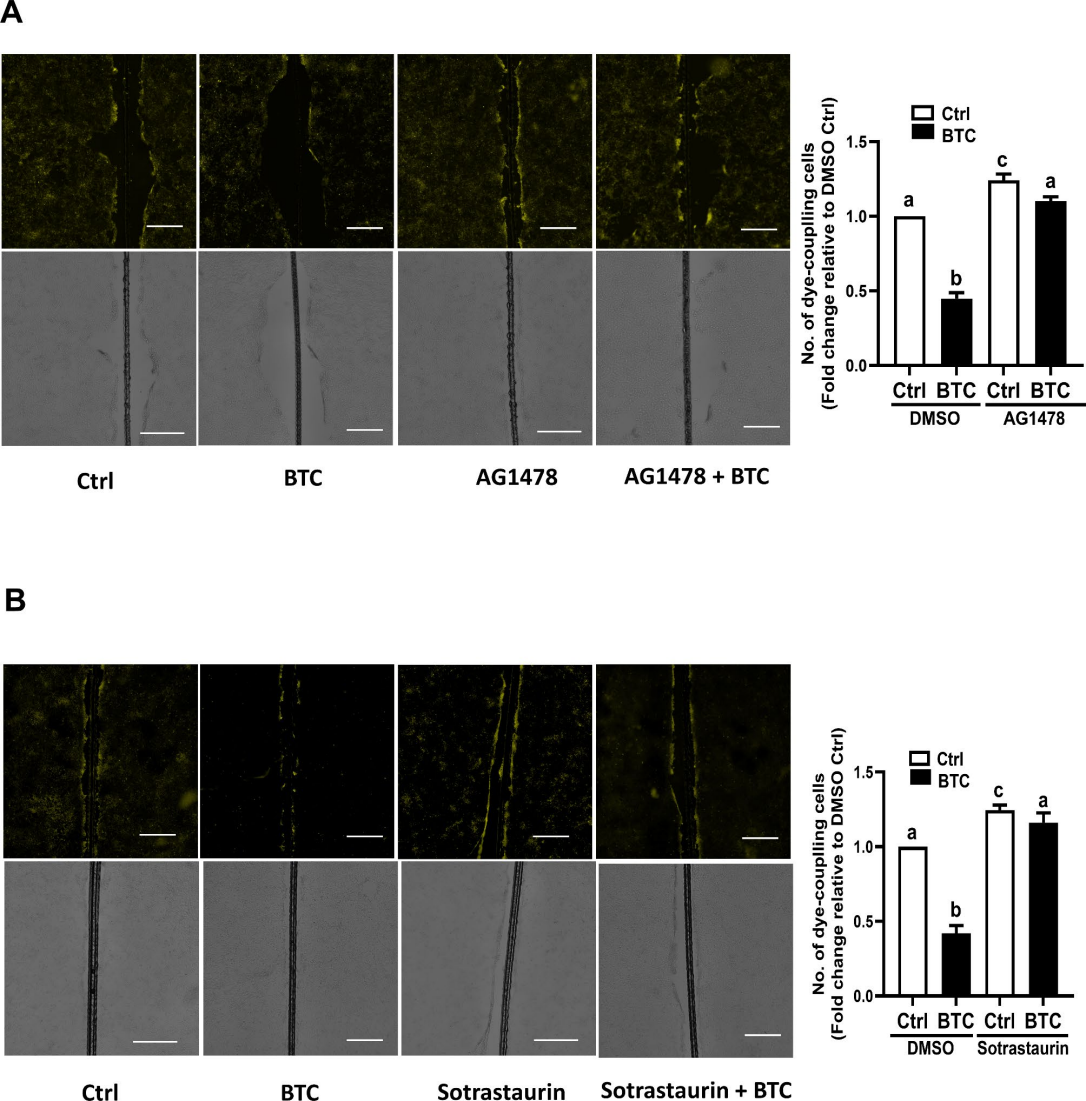
Effects of AG1478 and sotrastaurin on BTC-induced decreases in gap junction intercellular communication (GJIC) in SVOG cells. (A and B) SVOG cells were pretreated with DMSO, 10 µM AG1478 (A) or 10 µM sotrastaurin (B) for 40 min, and the cells were then treated with 50 ng/ml BTC for an additional 15 min. GJIC activity was determined by measuring the transfer of Lucifer yellow fluorescent dye between cells. The images were captured utilizing a fluorescence microscope (top panel). The corresponding phase contrast micrographs are shown in the bottom panel. The amount of dye transferred from one cell to its connected neighbor is dependent on the number of gap junctions that are coupled. The GJIC was estimated as an area where Lucifer yellow diffuses during a certain period away from the scrape line to the furthest extent of the dye-coupled cells. The expression or fluorescence intensities were quantified using ImageJ 1.42 software (NIH, U.S.A.). The experiments were performed in triplicate. Fluorescence intensity was calculated as fluorescence intensity of the treatment group divided by the fluorescence intensity of the control group. The image background was subtracted by ImageJ software. The scale bar represents 25 μm. Magnification, 200×. The results are expressed as the mean ± SEM of at least three independent experiments. Values marked with different letters were significantly different (P < 0.05)

To investigate the effect of BTC-induced phosphorylation of Cx43 on gap junction intercellular communication between hGL cells, a scrape-loading and dye transfer assay was applied. As shown in Fig. 7A, the number of lucifer yellow dye-coupled cell layers on either side of the scrape significantly decreased after BTC treatment for 15 min. Intriguingly, this effect was reversed by pretreatment for 40 min with either 10 µM AG1478 or 10 µM sotrastaurin (Fig. 7A and B). These results indicate that EGFR and PKC are involved in the BTC-induced decrease in GJIC in SVOG cells.

## Discussion

Emerging evidence suggests that the first step of oocyte meiotic resumption is the cessation of GJIC between an oocyte and surrounding cumulus cells [31]. The disruption of GJIC is mainly regulated by the phosphorylation of Cx43, leading to a decline in cGMP transported from cumulus cells or granulosa cells to an oocyte [32]. The decrease in cGMP concentration in an oocyte stimulates the activity of PDE3A, which further reduces the intracellular cAMP level [33]. GJIC activity can be regulated by altering Cx43 expression or phosphorylation of the Cx43 protein. Our previous studies revealed that members of the transforming growth factor β (TGF-β) superfamily differentially modulated the GJIC activity by regulating the expression of Cx43 in hGL cells [34]. Specifically, TGF-β1 enhanced GJIC activity by upregulating Cx43 expression [35], whereas BMPs suppressed GJIC activity by downregulating Cx43 expression in hGL cells [36–39]. In this study, we presented the first data showing that BTC treatment significantly decreased GJIC activity in hGL cells, and this effect is most likely mediated by the BTC-induced increase in Cx43 phosphorylation but not a change in Cx43 expression. In contrast to our results, EGF has been shown to regulate GJIC activity by upregulating Cx43 expression in porcine granulosa cells [40]. These results indicate that several intrafollicular factors work together to regulate GJIC activity through different molecular mechanisms, which results in oocyte meiotic resumption in the periovulatory phase.

In terms of functional receptors, BTC exerts its cellular effects by binding to different combinations of ErbB heterodimers, including EGFR and ErbB4 homodimers [9, 10]. A comprehensive understanding of the corresponding receptor involved in ligand-induced cellular activities is important benefit the development of therapeutic strategies of related diseases. To determine the receptor involved in the BTC-induced cellular activities in hGL cells, we used a dual inhibition approach. Specifically, the addition of the EGFR kinase inhibitor AG1478 completely abolished the BTC-induced increase in Cx43 phosphorylation, distribution of phosphorylated Cx43, and decrease in GJIC activity. Additionally, knocking down EGFR completely abolished the BTC-induced increase in Cx43 phosphorylation, whereas knocking down ErbB4 exerted no similar effect. These results indicate that EGFR but not ErbB4 was the main functional receptor that mediated BTC-induced activities in hGL cells. Consistent with our results, previous studies have shown a critical role for EGFR in the regulation of oocyte maturation and embryo development in various mammalian species [41–44].

Studies have shown that the phosphorylated Cx43 protein was activated by either the MAPK or PKC signaling pathway in rat liver epithelial cells [45]. In mouse embryonic stem cells, EGF enhanced cell proliferation by inducing the phosphorylation of Cx43 via the Ca2+/PKC, p44/42, and p38 MAPK pathways [46]. In this regard, different signaling pathways may induce differential phosphorylation sites (serine is the most common amino acid phosphorylated) in the Cx43 protein in response to EGF stimulation. For instance, the phosphorylation of Cx43 at Ser262 and Ser279/282 was induced by the activation of the MAPK pathway, whereas the phosphorylation of Cx43 at Ser368 was induced by the PKC signaling pathway in mouse embryonic stem cells [47]. In addition to EGF, other EGF family ligands have been shown to regulate phosphorylated Cx43-mediated gap junction communication activity. In granulosa cell-specific *Nrg1*-knockout mice, oocyte meiosis resumption occurs earlier than in wild-type mice, and most oocytes in Nrg1-deficient mice spontaneously resume meiosis after the MII stage, because of the previously unidentified phosphorylation of Cx43 at Ser368 induced by the protein kinase C (PKC) signaling pathway in cumulus cells [48]. In the present study, we demonstrated that BTC activated PKC, and this effect was abolished by pretreatment with either AG1478 or sotrastaurin. Furthermore, we showed that pretreatment with sotrastaurin completely abolished the BTC-induced increase in Cx43 phosphorylation at Ser368. However, pretreatment with U0126 had no effect. These findings indicate that PKC signaling (but not MAPK signaling) is the main pathway that mediates the BTC-induced increase in Cx43 phosphorylation at Ser368 in hGL cells.

There are some limitations that exist in this study. Although we have confirmed that BTC can induce the phosphorylation of Cx43 at Ser368, which led to a decrease in GJIC activity in both primary and immortalized hGL cells, whether other EGF family members can regulate the phosphorylation of Cx43 remains undetermined. Additionally, our results were obtained from the in vitro experiments. These results were generated from the cell culture system using both primary and immortalized hGL cells. Specifically, all these cells were derived from the follicular fluid of IVF patients after ovulation induction procedures, which have been exposed to pharmacological doses of gonadotrophins and human chorionic gonadotrophins. Compared to the granulosa cells in growing follicles, these luteinized granulosa cells have different cell activities and expression levels of the related membrane receptors. Furthermore, the established in vitro system may be not relevant to the in vivo microenvironment of the ovarian follicles. Future in vivo studies performed using animal models or relevant clinical samples will provide more information regarding the functional roles of BTC in regulating the phosphorylation of Cx43 and the related GJIC in hGL cells.

## Conclusions

In summary, we demonstrated that one of the EGF family members, BTC, promptly induced the phosphorylation of connexin 43 at Ser368, and this led to decreased GJIC activity in both primary and immortalized hGL cells. Additionally, BTC-induced cellular activities were most likely driven by the EGFR-mediated PKC-dependent signaling pathway in hGL cells. Given the important role played by EGF family factors in oocyte maturation and the high expression level of Cx43 in human granulosa cells, a comprehensive understanding of the underlying molecular mechanisms by which EGF family growth factors regulate GJIC activity will yield information useful for developing target strategies for women with ovulation disorders.

## Methods

### Culture of simian virus 40 large T antigen–immortalized human granulosa (SVOG) cells

SVOG cells were used in the present study to evaluate the influences of BTC treatment. First, a hemocytometer was used to count cells, and 0.04% trypan blue was utilized to assess cell viability. Then, the cells were seeded in 6-well plates or dishes and cultured in a humidified atmosphere containing 5% CO_2_ and 95% air at 37 °C. The cells were cultured in Dulbecco’s modified Eagle’s medium/nutrient mixture F-12 Ham (DMEM/F-12; Sigma‒Aldrich Corp., Oakville, ON) supplemented with 10% charcoal/dextran-treated fetal bovine serum (HyClone, Logan, UT), 100 µg/ml streptomycin sulfate (Invitrogen, Life Technologies), 100 U/ml penicillin (Invitrogen, Life Technologies, NY), and GlutaMAX (1X, Invitrogen, Life Technologies). The medium for SVOG cell culture was changed every other day. Subsequently, serum-free DMEM/F-12 was used to starve cells for 12 h before BTC treatment.

### Preparation and culture of primary human granulosa-lutein (hGL) cells

Primary hGL cells were obtained with informed patient consent following approval from the University of British Columbia Ethics Board. Two controlled ovarian stimulation protocols for in vitro fertilization patients were utilized: (1) one involved luteal-phase nafarelin acetate (Synarel, Pfizer, Kirkland, Quebec, Canada) and (2) another was based on follicular phase GnRH antagonist (Ganirelix; Merck Canada) down regulation. Gonadotropin stimulation was initiated on menstrual cycle Day 2 by administration of human menopausal gonadotropin (hMG; Menopur, Ferring, Canada) and recombinant FSH (Puregon, Merck, Canada). Based on follicle size, human chorionic gonadotropin was administered 34–36 h before oocyte retrieval. Granulosa cells were purified by density centrifugation of follicular aspirates obtained from women undergoing oocyte retrieval as previously described [49]. Primary hGL cells collected from individual females were cultured separately. Purified hGL cells were seeded in 6-well plates in the same culture environment and culture medium that was used with the SVOG cell line.

### Antibodies and reagents

Polyclonal rabbit anti-connexin 43 (^#^3512) (1:1000) and polyclonal rabbit anti-phospho-connexin 43 (Ser368) (D6W8P) (^#^52,559), polyclonal rabbit anti-EGFR (#2232) (1:1000) and monoclonal rabbit anti- HER4/ErbB4 (111B2) (#4795) (1:1000) antibodies were obtained from Cell Signaling Technology (Beverly, MA). A monoclonal mouse anti-α-tubulin (B-5-1-2) (sc-23,948) (1:3000) antibody was obtained from Santa Cruz Biotechnology (Santa Cruz, CA). Horseradish peroxidase-conjugated goat anti-rabbit IgG and goat anti-mouse IgG were obtained from Bio-Rad Laboratories (Hercules, CA). E. coli-derived recombinant human betacellulin (Asp32-Tyr111) was obtained from R&D Systems (Minneapolis, MN). AG 1478 was obtained from Sigma‒Aldrich Corp. U0126 was obtained from Calbiochem. Sotrastaurin (AEB071) and a PKC-theta inhibitor were obtained from Selleckchem.

### Reverse transcription and real-time quantitative PCR (RT‒qPCR)

Cells were washed with cold phosphate-buffered saline (PBS), and total RNA was extracted with TRIzol reagent (Invitrogen) according to the manufacturer’s instructions. RNA (2 µg) was reverse-transcribed into first-strand cDNA with random primers and MMLV reverse transcriptase (Promega, Madison, WI). Each 20-µl qPCR consisted of 1X SYBR Green PCR Master Mix (Applied Biosystems, Foster City, CA), 20 ng of cDNA and 250 nM of each primer. The primers used were *GJA1* (*Cx43*), 5’- TAC CAA ACA GCA GCG GAG TT -3’ (sense) and 5’- TGG GCA CCA CTC TTT TGC TT -3’ (antisense); and *glyceraldehyde-3-phosphate dehydrogenase* (*GAPDH*), 5’- ATG GAA ATC CCA TCA CCA TCT T -3’ (sense) and 5’- CGC CCC ACT TGA TTT TGG − 3’ (antisense). qPCR was performed on an Applied Biosystems 7300 Real-Time PCR System equipped with a 96-well optical reaction plate (Applied Biosystems). The specificity of each assay was validated by dissociation curve analysis and agarose gel electrophoresis of the PCR products. Assay performance was validated by evaluating the amplification efficiencies on the basis of the means of calibration curves and by ensuring that the plot of the log input amount vs. ^Δ^Cq (also known as ^Δ^CT) had a slope < |0.1|. The PCR parameters were 50 °C for 2 min, 95 °C for 10 min and 40 cycles of 95 °C for 15 s and 60 °C for 1 min. Three separate experiments were performed with different cultures, and each sample was assayed in triplicate. A mean value was used for the determination of mRNA levels via the comparative Cq (2^–ΔΔCq^) method with *GAPDH* used as the reference gene.

### Western blot analysis

After treatment, the cells were washed with cold PBS 3 times and lysed in lysis buffer (Cell Signaling) containing a protease inhibitor cocktail (Sigma‒Aldrich). Extracts were centrifuged at 20,000 x *g* for 10 min at 4 °C to remove cell debris. A DC Protein Assay (Bio-Rad Laboratories) was used to quantify the protein concentrations. Equal amounts of protein were separated by 10% sodium dodecyl sulfate polyacrylamide gel electrophoresis (SDS‒PAGE) and transferred from the gels to polyvinylidene fluoride membranes. The membranes were blocked with 5% nonfat dried milk in Tris-buffered solution containing 0.05% Tween 20 at room temperature for 1 h and incubated with the relevant primary antibodies at 4 °C overnight. After washing with TBS, the membranes were incubated with a peroxidase conjugated secondary antibody (Bio-Rad) at room temperature for 1 h. An enhanced chemiluminescence substrate or a SuperSignal West Femto Chemiluminescence Substrate (Pierce, Rockford, IL) was utilized as needed. The membranes were exposed to CL-XPosure film (Thermo Fisher, Waltham, MA) for detection of the immunoreactive bands. Stripping buffer (50 mM Tris-HCl, pH 7.6; 10 mmol/l β-mercaptoethanol; and 1% SDS) was used to strip membranes at 50 °C for 30 min, and the membranes were reprobed with a rabbit anti-Cx43 antibody as the loading control. In protein electrophoresis, the typical pattern of Cx43 (GJA1) are the two bands detectable in Western blot analysis, which are referred to as upper band and lower band [50]. The upper band resembles the non-phosphorylated more immature Cx43 (GJA1), while the lower band represents the phosphorylated Cx43 (GJA1), which is present in functional gap junction plaques [51]. Band intensities of western blot staining were measured and quantitatively analyzed using ImageJ 1.42 (NIH, U.S.A.). The experiments were performed in triplicate. Quantification graphs reflect Integrated Density Values of the treatment group divided by the Integrated Density Values of control group.

### Small interfering RNA (siRNA) transfection

Cells were precultured to 50% confluence in antibiotic-free DMEM/F12 with 10% charcoal/dextran-treated fetal bovine serum and then transfected with 25 nM EGFR-targeting siRNA (ON-TARGET*plus* SMARTpool, Catalog ID: L-003114-00-0020), 25 nM ErbB4-targeting siRNA (ON TARGET*plus* SMARTpool, Catalog ID: L-003128-00-0020), or 25 nM control siRNA (ON-TARGET*plus* Nontargeting Pool, Catalog ID: D-001810-01-20) (Dharmacon, Invitrogen) for 48 h using Lipofectamine RNAiMAX (Invitrogen). The efficiency of target gene was confirmed by Western blotting.

### Immunofluorescence staining

Cells were plated on glass cover slips, grown to 80% confluence, and fixed with methanol at -20 °C for 30 min. After they were washed with cold PBS three times, the cover slips were blocked with Protein Block Serum-free Buffer (Dako, Mississauga, Ontario, Canada) for 1 h and then incubated with a rabbit anti-phospho-Cx43 antibody (1:100 diluted in Protein Block Serum-free Buffer) at 4 °C overnight. After the cover slips were washed with cold PBS three times, they were incubated with a secondary antibody (Alexa Fluor 555 donkey anti-rabbit IgG, Life Technologies) at room temperature for 1 h. After the cover slips were washed with cold PBS three times, they were counterstained with Prolong™ Gold anti-fading reagent containing DAPI (Thermo) and imaged with a Zeiss Axiophot fluorescence microscope equipped with a digital camera (Q Imaging, Burnaby, BC, Canada). Quantification of the fluorescence intensities were quantified using ImageJ 1.42 (NIH, U.S.A.). The experiments were performed in triplicate. Fluorescence intensity was calculated as fluorescence intensity of the treatment group divided by the fluorescence intensity of control group. The image background was subtracted by ImageJ software.

### Protein kinase C (PKC) activity measurement

After treatment, the cells were washed with cold PBS 3 times and lysed in lysis buffer (Cell Signaling) containing a protease inhibitor cocktail (Sigma‒Aldrich). Extracts were centrifuged.

at 20,000 x *g* for 10 min at 4 °C to remove cell debris. A DC Protein Assay (Bio-Rad Laboratories) was used to quantify protein concentrations. A total of 0.125 µg of each extract was used for the PKC kinase assay, which was performed with a PKC Kinase Activity Assay Kit (Abcam) according to the manufacturer’s protocol. The kit is based on an ELISA (enzyme-linked immune-absorbent assay), in which a specific peptide is a substrate for PKC and a polyclonal antibody recognizes the phosphorylated form of the substate. The absorbance was measured at 450 nm with a microplate reader to determine PKC activity.

### Scrape-loading and dye transfer assay

To date, the scrape-loading and dye transfer assay is the most sensitive assay to evaluate intercellular communication [52]. This technique has been widely used to elucidate the qualitative and quantitative presence or absence of GJIC. Lucifer yellow (MW 457.2) is the most popular used dye with a high fluorescence efficiency. After introduction of Lucifer yellow into living cells, it cannot diffuse through intact cell membranes; however, because of its low molecular weight, it can be transferred to adjacent cells via intact gap junctions. To determine the regulatory effect of BTC on human granulosa cell GJIC activity, we performed scrape-loading and dye transfer assays. After the cells were cultured to 90% confluence, they were pretreated with inhibitors or DMSO and then treated with 50 ng/ml BTC for an additional 15 min. The cells were then washed with PBS and scraped with a surgical blade prior to the addition of fluorescent dye (0.5% Lucifer yellow CH, a potassium salt, Life Technology). Next, the cells were incubated for 5 min and washed with PBS three times to completely remove background fluorescence. Then, the cells were fixed with 4% paraformaldehyde and imaged with a Zeiss Axiophot fluorescence microscope equipped with a digital camera. The amount of dye transferred from one cell to its connected neighbor is dependent on the number of gap junctions that are coupled. The GJIC was estimated as an area that the Lucifer yellow diffuses during a certain period away from the scrape line to the furthest extent of the dye-coupled cells. The expression or fluorescence intensities were quantified using ImageJ 1.42 software (NIH, U.S.A.). The experiments were performed in triplicate. All fluorescence images were acquired using an inverted microscope equipped with fluorescence excitation. The microscope is equipped with both excitation and emission filter wheels, filter sets E4: bandpass filter (BP 436/7nm); long-pass filter (LP 490 mm). Fluorescence intensity was calculated as fluorescence intensity of the treatment group divided by the fluorescence intensity of the control group. The image background was subtracted by ImageJ software.

### Statistical analysis

The results were analyzed by one-way ANOVA followed by Tukey’s multiple comparison test in PRISM software (GraphPad Software, Inc., San Diego, CA). The results are presented as the mean ± SEM of at least three independent experiments. The data were considered significantly different when *P* < 0.05. Different letters indicate significant a difference (*p* < 0.05). In detail, if the letters on two columns are different (E.g., “a” vs. “b” or “b” vs. “c”), it means that the difference between the two groups is significant, on the other hand, if the letters on the column of two groups are the same (E.g., “a” vs. “a” or “b” vs. “b”), it means there is no significant difference between two groups.

## Data Availability

All data generated or analyzed during this study are included in this published article.

## Abbreviation

AREG: Amphiregulin
BTC: Betacellulin
COC: Cumulus-oophorus complex
Cx43: Connexin 43
EREG: Epiregulin
DMEM/F-12: Dulbecco’s modified Eagle’s medium/nutrient mixture F-12 Ham
EGF: Epidermal growth factor
EGFR: Epidermal growth factor receptor
ErbB4: Receptor tyrosine-protein kinase 4
FSH: Follicle-stimulating hormone
GAPDH: Glyceraldehyde-3-phosphate dehydrogenase
GJIC: Gap junction intercellular communication
GVBD: Germinal vesicle breakdown
hGL: Human granulosa-lutein
IVF: In vitro fertilization
LH: Luteinizing hormone
MMLV: Moloney Murine Leukemia Virus
PBS: Phosphate-buffered saline
PKC: Protein kinase C
PVDF: Polyvinylidene fluoride
RT-qPCR: Reverse transcription quantitative real-time polymerase chain reaction
siRNA: Small interfering RNA
SDS-PAGE: Sodium dodecyl sulfate polyacrylamide gel electrophoresis
TBS: Tris-buffered saline
SVOG cells: Immortalized human granulosa cells
TGF-β: Transforming growth factor-β
